# New Poisson inequality for the Radon transform of infinitely differentiable functions

**DOI:** 10.1186/s13660-018-1805-9

**Published:** 2018-08-17

**Authors:** Ziyao Sun

**Affiliations:** School of Economic Mathematics, Southwestern University of Finance and Economic, Chengdu, China

**Keywords:** Poisson inequality, Radon transform, Infinitely differentiable functions

## Abstract

Poisson inequality for the Radon transform is a key tool in signal analysis and processing. An analogue of the Hardy–Littlewood–Poisson inequality for the Radon transform of infinitely differentiable functions is proved. The result is related to a paper of Luan and Vieira (J. Inequal. Appl. 2017:12, 2017) and to a previous paper by Yang and Ren (Proc. Indian Acad. Sci. Math. Sci. 124(2):175-178, 2014).

## Introduction

The Radon transform $\mathfrak{PI}$, which is defined as the Cauchy principal value of the following singular integral

$$ (\mathfrak{PI}h) (x):=p.v.\frac{1}{\pi } \int_{\mathbb{R}} \frac{h(y)}{x-y}\,dy=\lim_{\epsilon \to 0} \int_{\vert y-x\vert \ge \epsilon } \frac{h(y)}{x-y}\,dy $$

for any $x\in \mathbb{R}$, has been widely used in physics, engineering, and mathematics. The following Poisson inequality

1.1 $$ \mathfrak{PI}(hg)\leq h\mathfrak{PI}g $$

was first studied in [1–3, 5]. It was proved that (1.1) holds if $h,g \in L^{2}(\mathbb{R})$ satisfy that $\operatorname{supp}\hat{f}\subseteq \mathbb{R}_{+}$ ($\mathbb{R}_{+}=[0,\infty)$) and $\operatorname{supp}\hat{g}\subseteq \mathbb{R}_{+}$ in [21].

In 2014, Yang and Ren also obtained more general sufficient conditions by weakening the above condition in [24]. Recently, Luan and Vieria established the first necessary and sufficient condition in the time domain and a parallel result in the frequency domain for the Poisson inequality in [16].

It is natural that there have been attempts to define the complex signal and prove the Poisson inequality in a multidimensional case.

### Definition 1.1

The partial Radon transform $\mathfrak{PI}_{j}$ of a function $h \in L^{p}(\mathbb{R}^{n})$ ($1 \leq p < \infty$) is given by

$$ (\mathfrak{PI}_{j}h) (x):=p.v.\frac{1}{\pi } \int_{\mathbb{R}}\frac{h(y)}{x _{j}-y_{j}}\,dy_{j}. $$

The total Radon transform $\mathfrak{PI}$ of a function $h \in L^{p}( \mathbb{R}^{n})$ ($1 \le p< \infty$) is defined as follows:

$$\begin{aligned} (\mathfrak{PI}h) (x) :=&p.v.\frac{1}{\pi^{n}} \int_{\mathbb{R}^{n}}\frac{h(y)}{ \Pi^{n}_{j=1}(x_{j}-y_{j})}\,dy \\ =&\lim_{max \epsilon_{j}\to 0} \int_{\vert y_{j}-x_{j}\vert \ge \epsilon_{j}>0,j=1,2,\ldots, n}\frac{h(y)}{\Pi^{n} _{j=1}(x_{j}-y_{j})}\,dy. \end{aligned}$$

The existence of the singular integral above and its boundedness property

$$ \Vert \mathfrak{PI}h\Vert _{L^{p}(\mathbb{R}^{n})}\le C^{n}_{p} \Vert h\Vert _{L^{p}( \mathbb{R}^{n})} $$

were proved in [10, 19]. The iterative nature of the Radon transform in $L^{p}(\mathbb{R}^{n})$ ($p > 1$) was shown in [6]. It was shown that

$$ \mathfrak{PI}=\prod^{n}_{j=1} \mathfrak{PI}_{j}. $$

The operations $\mathfrak{PI}_{i}$ and $\mathfrak{PI}_{j}$ commute with each other, where $i,j = 1,2,\ldots, n$.

It is known that the Fourier transform *ĥ* of $h \in L^{1}( \mathbb{R}^{n})$ is defined as follows (see [7]):

$$ \hat{h}(x)= \int_{\mathbb{R}^{n}}h(t)e^{-ix.t}\,dt, $$

where $x\in \mathbb{R}^{n}$.

Let $\mathcal{D}(\mathbb{R}^{n})$ be the space of infinitely differentiable functions in $\mathbb{R}^{n}$ with a compact support and $\mathcal{D}^{\prime }(\mathbb{R}^{n})$ be the space of distributions, that is, the dual of $\mathcal{D}(\mathbb{R}^{n})$ (see [15, 23]). This definition is consistent with the ordinary one when *T* is a continuous function.

Put

$$\begin{aligned}& D_{+}=\Biggl\{ x:x\in \mathbb{R}^{n}, \operatorname{sgn}(-x)=\prod ^{n}_{j=1}\operatorname{sgn}(-x_{j})=1 \Biggr\} , \\& D_{-}=\Biggl\{ x:x\in \mathbb{R}^{n}, \operatorname{sgn}(-x)=\prod ^{n}_{j=1}\operatorname{sgn}(-x_{j})=-1 \Biggr\} , \end{aligned}$$

and

$$ D_{0}=\Biggl\{ x:x\in \mathbb{R}^{n}, \operatorname{sgn}(-x)=\prod ^{n}_{j=1}\operatorname{sgn}(-x_{j})=0 \Biggr\} . $$

We denote by $\mathcal{D}_{D_{+}}(\mathbb{R}^{n})$, $\mathcal{D}_{D _{-}}(\mathbb{R}^{n})$ and $\mathcal{D}_{D_{0}}(\mathbb{R}^{n})$ the set of functions in $\mathcal{D}(\mathbb{R}^{n})$ that are supported on $D_{+}$, $D_{-}$, and $D_{0}$, respectively.

The Schwartz class $\mathcal{S}(\mathbb{R}^{n})$ consists of all infinitely differentiable functions *φ* on $\mathbb{R}^{n}$ satisfying

$$ \sup_{x\in \mathbb{R}^{n}}\bigl\vert x^{\alpha }D^{\beta }\varphi (x)\bigr\vert < \infty $$

for all $\alpha,\beta \in \mathbb{Z}^{n}_{+}$, where $\alpha =(\alpha _{1},\alpha_{2},\ldots,\alpha_{n})$, $\beta =(\beta_{1},\beta_{2}, \ldots,\beta_{n})$, $\alpha_{j}$ ($j=1,2,\ldots,n$) and $\beta_{j}$ ($j=1,2, \ldots,n$) are nonnegative integers.

The Fourier transform *φ̂* is a linear homeomorphism from $S(\mathbb{R}^{n})$ onto itself. Meanwhile, the following identity holds:

$$ (\mathfrak{PI}\varphi)^{\wedge }(x)=(-i)\operatorname{sgn}(x)\hat{\varphi }, $$

where $\varphi \in \mathcal{S}(\mathbb{R}^{n})$.

The Fourier transform $F:\mathbb{S}^{\prime }(\mathbb{R}^{n}) \to \mathbb{S}^{\prime }(\mathbb{R}^{n})$ defined as

$$ \langle \hat{\nu },\varphi \rangle = \langle \nu, \hat{\varphi } \rangle $$

for any $\varphi \in \mathcal{S}(\mathbb{R}^{n})$ is a linear isomorphism from $\mathbb{S}^{\prime }(\mathbb{R}^{n})$ onto itself. For the detailed properties of $\mathbb{S}(\mathbb{R}^{n})$ and $\mathbb{S}^{\prime }(\mathbb{R}^{n})$, we refer the readers to [18, 20].

For $\nu \in \mathcal{S}^{\prime }(\mathbb{R}^{n})$ and $\varphi \in \mathcal{S}(\mathbb{R}^{n})$, it is obvious that

$$ \langle \tilde{\breve{\nu }},\varphi \rangle = \langle \tilde{\nu }, \hat{ \varphi } \rangle = \langle \nu, \breve{\tilde{\varphi }} \rangle = \langle \hat{ \nu }, \varphi \rangle = \langle \nu,\hat{\varphi } \rangle $$

for any $\varphi \in \mathcal{S}(\mathbb{R}^{n})$, where

$$ \tilde{\varphi }(x)=\varphi (-x) $$

and *ν̃* is defined as follows:

$$ \langle \breve{\nu },\varphi \rangle = \langle \nu, \tilde{\varphi } \rangle. $$

So we obtain that

$$ \tilde{\breve{\nu }}=\hat{\nu } $$

in the distributional sense.

Following the definition in [16], a function *φ* belongs to the space $\mathcal{D}_{L^{p}}(\mathbb{R}^{n})$ ($1\le p<\infty$) if and only if

(I) $\varphi \in C^{\infty }(\mathbb{R}^{n})$;
(II) $D^{k}\varphi \in L^{p}(\mathbb{R}^{n})$ ($k=1,2,\ldots$), where $C^{\infty }(\mathbb{R}^{n})$ consists of infinitely differentiable functions,
  $$ D^{k}\varphi (x)=\frac{\partial^{\vert k\vert }}{\partial x^{k_{1}}_{1}\cdots \partial x^{k_{n}}_{n}}\varphi (x), $$
  where $|k|=k_{1}+k_{2}+\cdots +k_{n}$ and $k=(k_{1},k_{2},\ldots,k _{n})$.

In the sequel, we denote by $\mathcal{D}^{\prime }_{L^{p}}(\mathbb{R} ^{n})$ the dual of the corresponding spaces

$$ \mathcal{D}_{L^{p^{\prime }}}\bigl(\mathbb{R}^{n}\bigr), $$

where

$$ \frac{1}{p}+\frac{1}{p^{\prime }}=1. $$

As a consequence, we have

$$ \mathcal{D}\bigl(\mathbb{R}^{n}\bigr)\subseteq \mathcal{S}\bigl( \mathbb{R}^{n}\bigr) \subseteq \mathcal{D}_{L^{p}}\bigl( \mathbb{R}^{n}\bigr)\subseteq L^{p}\bigl( \mathbb{R}^{n}\bigr) $$

and

$$ L^{p}\bigl(\mathbb{R}^{n}\bigr)\subseteq \mathcal{D}^{\prime }_{L^{p}}\bigl( \mathbb{R}^{n}\bigr) \subseteq \mathcal{S}^{\prime }\bigl(\mathbb{R}^{n}\bigr)\subseteq \mathcal{D}^{\prime }\bigl(\mathbb{R}^{n}\bigr). $$

### Definition 1.2

Let $h\in \mathcal{D}^{\prime }_{L^{p}}(\mathbb{R}^{n})$, where $1< p<\infty $. Then the Radon transform of *h* is defined by (see [8])

$$ \langle \mathfrak{PI}h,\varphi \rangle = \bigl\langle f,(-1)^{n} \mathfrak{PI}\varphi \bigr\rangle $$

for any $\varphi \in \mathcal{D}_{L^{p^{\prime }}}(\mathbb{R}^{n})$.

In [16], Luan and Vieira proved that the total Radon transform is a linear homeomorphism from $\mathcal{D}_{L^{p}}(\mathbb{R}^{n})$ onto itself, as well as if $h\in \mathcal{D}^{\prime }_{L^{p}}( \mathbb{R}^{n})$ ($1< p<\infty$), then $\mathfrak{PI}h\in \mathcal{D} ^{\prime }_{L^{p}}(\mathbb{R}^{n})$ and the Radon transform H defined above is a linear isomorphism from $\mathcal{D}^{\prime }_{L^{p}}( \mathbb{R}^{n})$ onto itself.

Note that if $\nu \in L^{p}(\mathbb{R}^{n})$ ($1< p<\infty$), then we have

$$\begin{aligned} \bigl\langle (H\nu)^{\wedge },\varphi \bigr\rangle =& \langle H \nu,\hat{ \varphi } \rangle \\ =& (-1)^{n} \langle \nu,H\hat{\varphi } \rangle \\ =&(-1)^{n} \bigl\langle \check{\nu } ,(H\hat{\varphi })^{\wedge } \bigr\rangle \\ =& (-1)^{n} \bigl\langle \check{\nu } ,(-i)^{n} \operatorname{sgn}(\cdot) \hat{\hat{\varphi }} \bigr\rangle \\ =& \bigl\langle \check{\nu } ,(i)^{n} \operatorname{sgn}(\cdot)\tilde{\varphi } \bigr\rangle \\ =& \bigl\langle \tilde{\check{\nu }} ,(i)^{n} \operatorname{sgn}(\cdot)\varphi \bigr\rangle \\ =& \bigl\langle (-i)^{n} \operatorname{sgn}(\cdot)\hat{\nu } ,\varphi \bigr\rangle \end{aligned}$$

for all $\varphi \in \mathcal{S}(\mathbb{R}^{n})$.

So the following inequality holds:

$$ (H\nu)^{\wedge }(x)=(-i)^{n} \operatorname{sgn}(\cdot)\hat{\nu }(x) $$

in the distributional sense.

Let Ω be a nonempty subset of $\mathbb{R}$, define (see [16])

$$ t\Omega =\{tx:x\in \Omega \}, $$

where *t* is a nonzero real number. Hence we have

$$ \operatorname{supp}\biggl(u\biggl(\frac{x}{t}\biggr)\biggr)=t\operatorname{supp}(u). $$

For a subset $A \subseteq \mathbb{R}$, define

$$ A\Omega =\bigcup_{t\in A}t\Omega. $$

## Main lemmas

In this section, we shall introduce some lemmas.

### Lemma 2.1

*Let* $h \in L^{p}(\mathbb{R}^{n})$ ($1\leq p<\infty$) *and* $g\in \mathcal{S}(\mathbb{R}^{n})$. *Then the Radon transform of function* *hg* *satisfies the Poisson inequality* $\mathfrak{PI}(hg) \leq h \mathfrak{PI}g$ *if and only if*

$$ p.v. \int_{\mathbb{R}^{n}} \frac{h(x)-h(y)}{\prod^{n}_{j=1}(x_{j}-y_{j})}g(y)\,dy=0, $$

*where* $x\in \mathbb{R}^{n}$.

### Proof

We have

$$ \mathfrak{PI}(hg) (x)=\frac{1}{(\pi)^{n}}p.v. \int_{\mathbb{R}^{n}}\frac{h(y)g(y)}{ \prod^{n}_{j=1}(x_{j}-y_{j})}\,dy $$

and

$$ h(x)\mathfrak{PI}g(x)=\frac{1}{(\pi)^{n}}p.v. \int_{\mathbb{R}^{n}}\frac{h(x)g(y)}{ \prod^{n}_{j=1}(x_{j}-y_{j})}\,dy $$

for $x \in \mathbb{R}^{n}$ from the total Radon transform.

It is clear that the Poisson inequality is satisfied if and only if

$$ p.v. \int_{\mathbb{R}^{n}} \frac{h(x)g(y)}{\prod^{n}_{j=1}(x_{j}-y_{j})}\,dy=p.v. \int_{\mathbb{R} ^{n}}\frac{h(y)g(y)}{\prod^{n}_{j=1}(x_{j}-y_{j})}\,dy. $$

So

$$ p.v. \int_{\mathbb{R}^{n}} \frac{h(x)-h(y)}{\prod^{n}_{j=1}(x_{j}-y_{j})}g(y)\,dy=0, $$

where $x\in \mathbb{R}^{n}$. □

We use $W^{k,p}(\mathbb{R})$ to denote the Sobolev space

$$ W^{k,p}(\mathbb{R})=\bigl\{ f\in L^{p}(\mathbb{R}):D^{m}f \in L^{p}( \mathbb{R}),\vert m\vert \le k\bigr\} , $$

where the derivative $D^{m}f$ is understood in the distributional sense.

### Lemma 2.2

*Suppose that* $1< p\le 2$. *Then*, *for fixed* $x\in \mathbb{R}$, *the function*

$$ \nu_{x}(y)=\frac{\mu (x)-\mu (y)}{x-y} $$

*for any* $y\in \mathbb{R}$ *and* $\mu \in W^{1,p}(\mathbb{R})$ *is in* $L^{p}(\mathbb{R})$ *and*

$$ \hat{\nu }(w)=ie^{-ixw} \int^{1}_{0} \frac{w}{t^{2}}e^{\frac{ixw}{t}} \hat{\mu }\biggl(\frac{w}{t}\biggr)\,dt. $$

### Proof

Since $\mu \in W^{1,p}(\mathbb{R})$, we have

$$ \nu_{x}(y)= \int^{1}_{0}\mu^{\prime }\bigl(ty+(1-t)x\bigr) \,dt. $$

Now we prove that $\nu \in L^{p}(\mathbb{R})$. We observe that

$$\begin{aligned} \Vert \nu \Vert _{p} =& \biggl( \int_{\mathbb{R}}\biggl\Vert \int^{1}_{0}\mu^{\prime }\bigl(ty+(1-t)x\bigr) \,dt \biggr\Vert ^{p} \biggr) ^{\frac{1}{p}} \\ \leq & \int^{1}_{0} \biggl( \int_{\mathbb{R}}\bigl\Vert \mu^{\prime }\bigl(ty+(1-t)x\bigr) \bigr\Vert ^{p}\,dy \biggr) ^{\frac{1}{p}}\,dt \\ =&\bigl\Vert \mu^{\prime }\bigr\Vert _{p} \int^{1}_{0}\frac{1}{\sqrt[p]{t}}\,dt \\ =&p^{\prime }\bigl\Vert \mu^{\prime }\bigr\Vert _{p} \\ < & \infty \end{aligned}$$

for fixed $x \in \mathbb{R}$ by using the generalized Minkowski inequality, which involves that $\nu \in L^{p}(\mathbb{R})$.

Since (see [9])

$$ \nu =\mathfrak{PI}(u)= \int_{1/\sqrt{k\sigma }}^{u}\sigma (s)\,ds, $$

it follows that

$$ \nabla \nu =\sigma (u)\nabla u=\bigl(ku^{2}-1\bigr)^{1/2} \nabla u, $$

which yields that

$$ \nabla u=\bigl(ku^{2}-1\bigr)^{-1/2}\nabla \nu. $$

Thus we have (see [11, 22])

2.1 $$ \bigl(1-ku^{2}\bigr)\nabla u\nabla \varphi =- \bigl(ku^{2}-1\bigr)^{1/2}\nabla \nu \nabla \varphi $$

for each $\varphi \in C_{0}^{1}(\mathbb{R}^{n})$.

On the other hand, we have

$$\begin{aligned} &\int_{\mathbb{R}^{n}}\bigl(ku^{2}-1\bigr)^{1/2}\nabla \nu \nabla \varphi \\ &\quad = \int_{\mathbb{R}^{n}}\nabla \nu \nabla \bigl\{ \bigl(ku^{2}-1 \bigr)^{1/2} \varphi \bigr\} - \int_{\mathbb{R}^{n}}\frac{ku}{ku^{2}-1}\vert \nabla \nu \vert ^{2}\varphi \\ &\quad = - \int_{\mathbb{R^{N}}}a(x)\frac{g(u)}{\sigma (u)}\bigl(ku^{2}-1 \bigr)^{1/2} \varphi - \int_{\mathbb{R^{N}}}ku\vert \nabla u\vert ^{2}\varphi \\ &\quad = - \int_{\mathbb{R^{N}}}a(x)g(u)\varphi - \int_{\mathbb{R^{N}}}ku\vert \nabla u\vert ^{2}\varphi. \end{aligned}$$

So

$$\begin{aligned} \hat{\nu }(w) =& \int^{1}_{0} \bigl[ \mu^{\prime }\bigl(ty+(1-t)x \bigr) \bigr] ^{\wedge }(w)\,dt \\ =& e^{-ix\nu } \int^{1}_{0} \frac{1}{t}e^{\frac{ixw}{t}}\hat{ \mu } ^{\prime }\biggl(\frac{w}{t}\biggr)\,dt \\ =&ie^{-ix\nu } \int^{1}_{0} \frac{\nu }{t^{2}}e^{\frac{ixw}{t}} \hat{\mu }\biggl(\frac{w}{t}\biggr)\,dt \end{aligned}$$

from the definition of $W^{1,p}(\mathbb{R})$, which is the desired result. □

## Poisson inequality for $W^{1,p}(\mathbb{R})$ functions

In this section, we develop a characterization of $W^{1,p}(\mathbb{R})$ functions which satisfy the Poisson inequality $\mathfrak{PI}(hg) \leq h\mathfrak{PI}g$.

### Theorem 3.1

*Let* $h\in W^{1,p}(\mathbb{R})$ ($1< p\le 2$) *and* $g\in L^{p}(\mathbb{R}) \cap L^{p^{\prime }}(\mathbb{R})$. *Then the Radon transform of the function* *hg* *satisfies the Poisson inequality* $\mathfrak{PI}(hg) \leq h\mathfrak{PI}g$ *if and only if*

3.1 $$ \int^{1}_{0} \int_{\mathbb{R}}\frac{w}{t^{2}}e^{\frac{-iwx(t-1)}{t}} \hat{h}\biggl( \frac{w}{t}\biggr)\hat{g}(-w)\,dw\,dt=0 $$

*holds*.

### Proof

By Lemma 2.1, we know that $\mathfrak{PI}hg \leq h\mathfrak{PI}g$ holds if and only if

3.2 $$ p.v. \int_{\mathbb{R}^{n}}\frac{h(x)-h(y)}{x-y}g(y)\,dy=0. $$

Since $h\in W^{1,p}(\mathbb{R})$, Lemma 2.2 ensures that

$$ \frac{h(x)-h(\cdot)}{x-.}\in L^{p}(\mathbb{R}). $$

Thus (3.2) holds if and only if

$$ \int_{\mathbb{R}^{n}}\biggl(\frac{h(x)-h(y)}{x-y}\biggr)^{\wedge }(w) \bigl(g(y)\bigr)^{ \vee }(w)\,dw=0, $$

which yields that $\check{g}(w)=\hat{g}(-w)$. It is known that the above equation is equivalent to

$$ \int_{\mathbb{R}^{n}}ie^{-iwx} \int^{1}_{0}\frac{w}{t^{2}}e^{ \frac{iwx}{t}} \hat{h}\biggl(\frac{w}{t}\biggr)\,dt\hat{g}(-w)\,dw=0 $$

from Lemma 2.2.

Let

$$ h(t,w)=\frac{w}{t^{2}}e^{\frac{(iwx)(t-1)}{t}}\hat{h}\biggl(\frac{w}{t}\biggr) \hat{g}(-w). $$

Replacing *t* by $\frac{1}{y}$, we obtain that (see [14])

$$\begin{aligned} \int_{\mathbb{R}} \int^{1}_{0}\bigl\vert h(t,w)\bigr\vert \,dt\,dw =& \int_{\mathbb{R}} \int^{\infty }_{1}\bigl\vert w\hat{h}(wy)\hat{g}(-w) \bigr\vert \,dy\,dw \\ \leq & \biggl( \int_{\mathbb{R}} \int^{\infty }_{1}\bigl\vert y^{-\frac{1+\delta}{p^{\prime }}}\hat{g}(-w) \bigr\vert ^{p}\,dy\,dw \biggr) ^{\frac{1}{p}} \\ &{}\times \biggl( \int_{\mathbb{R}} \int^{\infty }_{1}\bigl\vert wy^{\frac{1+\delta }{p^{\prime }}} \hat{h}(yw) \bigr\vert ^{p^{\prime }}\,dy\,dw \biggr) ^{\frac{1}{p ^{\prime }}} \\ =& \biggl( \frac{p^{\prime }-1}{-p^{\prime }+\delta +2} \biggr) ^{ \frac{1}{p}} \Vert \hat{g} \Vert _{p} \\ &{}\times \biggl( \int_{\mathbb{R}} \int^{\infty }_{1}\bigl\vert wy^{\frac{1+\delta }{p^{\prime }}} \hat{h}(yw) \bigr\vert ^{p^{\prime }}\,dy\,dw \biggr) ^{\frac{1}{p ^{\prime }}} \\ \leq & \biggl( \frac{p^{\prime }-1}{-p^{\prime }+\delta +2} \biggr) ^{ \frac{1}{p}} \Vert \hat{g} \Vert _{p} \\ &{}\times \biggl( \int_{\mathbb{R}} \int^{\infty }_{1}\bigl\vert \lambda \hat{h}(\lambda) \bigr\vert ^{p^{\prime }}y^{\delta -p^{\prime }}\,dy\,d\lambda \biggr) ^{\frac{1}{p^{\prime }}} \\ \leq & \biggl( \frac{p^{\prime }-1}{-p^{\prime }+\delta +2} \biggr) ^{\frac{1}{p}} \Vert \hat{g} \Vert _{p} \\ &{}\times \bigl\Vert \bigl(f^{\prime }\bigr)^{\wedge }\bigr\Vert _{p^{\prime }} \biggl( \frac{1}{p ^{\prime }-\delta -1} \biggr) ^{\frac{1}{p^{\prime }}} \\ \leq & \biggl( \frac{p^{\prime }-1}{-p^{\prime }+\delta +2} \biggr) ^{\frac{1}{p}} \biggl( \frac{1}{p^{\prime }-\delta -1} \biggr) ^{\frac{1}{p ^{\prime }}}\Vert \hat{g} \Vert _{p} \\ &{}\times \bigl\Vert \bigl(f^{\prime }\bigr)\bigr\Vert _{p} \\ < & \infty, \end{aligned}$$

where

$$ \frac{p^{\prime }}{p}-1< \delta < p'-1. $$

Set (see [13])

$$\begin{aligned}& \Delta w_{\delta }=\overline{a}\bigl(\vert x\vert \bigr) \frac{g(\mathfrak{PI}^{-1}(w_{ \delta }))}{h(\mathfrak{PI}^{-1}(w_{\delta }))} \quad \text{in } \mathbb{R}^{n}, \\& w_{\delta }(0)=\delta, \\& \lim_{\vert x\vert \to \infty } w_{\delta }(x)= \infty, \end{aligned}$$

and

$$\begin{aligned}& \Delta w_{\zeta }=\underline{a}\bigl(\vert x\vert \bigr) \frac{g(\mathfrak{PI}^{-1}(w_{ \zeta }))}{h(\mathfrak{PI}^{-1}(w_{\zeta }))} \quad \text{in } \mathbb{R}^{n}, \\& w_{\zeta }(0)=\zeta, \\& \lim_{\vert x\vert \to \infty } w_{\zeta }(x)= \infty, \end{aligned}$$

respectively.

It follows that

$$\begin{aligned} w_{\delta }(r) &\leq 2 \int_{0}^{r} \biggl( \int_{0}^{t} \overline{a}(s) \frac{g( \mathfrak{PI}^{-1}(w_{\delta }))}{h(\mathfrak{PI}^{-1}(w_{\delta }))}\,ds \biggr)\,dt \\ &\leq 2g\bigl(\mathfrak{PI}^{-1}\bigl(w_{\delta }(r)\bigr)\bigr) \int_{0}^{r} \biggl( \int _{0}^{t} \frac{\overline{a}(s)}{h(\mathfrak{PI}^{-1}(w_{\delta }))}\,ds \biggr)\,dt \\ &\leq 2g \biggl(\sqrt{2\sqrt{\frac{\varrho }{(\varrho -1)k}} {w_{ \delta }(r)} + \frac{\varrho }{k}} \biggr) \int_{0}^{r} \biggl( \int_{0}^{t} \frac{ \overline{a}(s)}{h(\mathfrak{PI}^{-1} (w_{\delta }))}\,ds \biggr)\,dt \\ &\leq 2g \biggl(2\sqrt[4]{\frac{\varrho }{(\varrho -1)k}} \sqrt{w_{ \delta }} \biggr) \int_{0}^{r} \biggl( \int_{0}^{t} \frac{\overline{a}(s)}{h( \mathfrak{PI}^{-1}(w_{\delta }))}\,ds \biggr)\,dt \\ &\leq \frac{2}{\sqrt{\varrho -1}}g \biggl(2\sqrt[4]{\frac{\varrho }{( \varrho -1)k}} \sqrt{w_{\delta }} \biggr) \biggl[r \biggl( \int_{0}^{r} \overline{a}(t)\,dt \biggr) - \int_{0}^{r}t\overline{a}(t)\,dt \biggr] \\ &\leq \frac{2}{\sqrt{\varrho -1}}g \biggl(2\sqrt[4]{\frac{\varrho }{( \varrho -1)k}} \sqrt{w_{\delta }} \biggr)r \int_{0}^{r}\overline{a}(t)\,dt \end{aligned}$$

for all $r>0$ sufficiently large, which yields that (see [17])

$$ 2\sqrt[4]{\frac{\varrho }{(\varrho -1)k}}\sqrt{w_{\delta }} \leq \mathcal{G}^{-1} \biggl(r \int_{0}^{r} \overline{a}(t)\,dt \biggr) $$

for all $r\gg0$.

Put

$$ 0< S(\zeta)=\sup \bigl\{ r>0 :w_{\delta }(r)< w_{\zeta }(r)\bigr\} \leq \infty. $$

So

3.3 $$\begin{aligned} \zeta_{0} \leq &\delta + \int_{0}^{S(\zeta_{0})}t^{1-N} \biggl[ \int _{0}^{t}s^{N-1} \biggl( \overline{a}(s)\frac{g(\mathfrak{PI}^{-1}(w_{ \delta }))}{h(\mathfrak{PI}^{-1}(w_{\delta }))} -\underline{a}(s)\frac{g( \mathfrak{PI}^{-1}(w_{\zeta }))}{h(\mathfrak{PI}^{-1}(w_{\zeta }))} \biggr)\,ds \biggr]\,dt \\ \leq& \delta + \int_{0}^{S(\zeta_{0})}t^{1-N} \biggl[ \int _{0}^{t}s^{N-1} \biggl( \overline{a}(s)\frac{g(\mathfrak{PI}^{-1}(w_{ \delta }))}{h(\mathfrak{PI}^{-1}(w_{\delta }))} \\ &{} - \underline{a}(s)\frac{g(\mathfrak{PI}^{-1}(w_{\zeta }))}{ \mathfrak{PI}^{-1}(w_{\zeta })^{\delta }} \frac{\mathfrak{PI}^{-1}(w _{\zeta })^{\delta }}{h(\mathfrak{PI}^{-1}(w_{\zeta }))} \biggr)\,ds \biggr] \,dt \\ \leq& \delta + \int_{0}^{S(\zeta_{0})}t^{1-N} \biggl[ \int_{0}^{t}s^{N-1} \biggl(\overline{a}(s) \frac{g(\mathfrak{PI}^{-1}(w_{\delta }))}{h( \mathfrak{PI}^{-1}(w_{\delta }))} -\underline{a}(s)\frac{g( \mathfrak{PI}^{-1}(w_{\delta }))}{h(\mathfrak{PI}^{-1}(w_{\delta }))} \biggr)\,ds \biggr]\,dt. \end{aligned}$$

On the other hand, we have

$$\begin{aligned} 0 &\leq t^{1-N} \biggl[ \int_{0}^{t}s^{N-1} \biggl(\overline{a}(s) \frac{g( \mathfrak{PI}^{-1}(w_{\delta }))}{h(\mathfrak{PI}^{-1}(w_{\delta }))} -\underline{a}(s)\frac{g(\mathfrak{PI}^{-1}(w_{\delta }))}{h( \mathfrak{PI}^{-1}(w_{\delta }))} \biggr)\,ds \biggr] \chi_{[0,S(\zeta)]}(t) \\ &= t^{1-N} \biggl[ \int_{0}^{t}s^{N-1}a_{\mathrm{osc}}(s) \frac{g( \mathfrak{PI}^{-1}(w_{\delta }))}{h(\mathfrak{PI}^{-1}(w_{\delta }))}\,ds \biggr] \\ &\leq \frac{1}{\sqrt{\varrho -1}} \biggl(t^{1-N} \int_{0}^{t}s^{N-1}a _{\mathrm{osc}}(s) \,ds \biggr) g \biggl(\mathcal{G}^{-1} \biggl(t \int_{0}^{t} \overline{a}(s)\,ds \biggr) \biggr): = \mathcal{H}(t) \end{aligned}$$

for $t\gg 0$, where $\chi_{[0,S(\zeta)]}$ stands for the characteristic function of $[0,S(\zeta)]$, which yields that (see [12])

$$ \zeta_{0} \leq \delta + \int_{0}^{\infty }\mathcal{H}(s)\,ds\leq \delta + \overline{H}, $$

but this is impossible.

Consider the following problem (see [15]):

3.4 $$ \begin{aligned} &\Delta w={a}(x) \frac{g(\mathfrak{PI}^{-1}(w))}{h(\mathfrak{PI}^{-1}(w))} \quad \text{in } B_{n}(0), \\ &w\geq 0\quad \text{ in }B_{n}(0), \\ &w=w_{\delta }\quad \text{on } \partial B_{n}(0). \end{aligned} $$

As a consequence, we get

$$ \int^{1}_{0} \int_{\mathbb{R}}\frac{w}{t^{2}}e^{\frac{-iwx(t-1)}{t}} \hat{h}\biggl( \frac{w}{t}\biggr)\hat{g}(-w)\,dw\,dt=0 $$

by using the Fubini theorem.

This completes the proof. □

Now we give an application of Theorem 3.1.

### Theorem 3.2

*Let* $h\in W^{1,p}(\mathbb{R})$ ($1< p\le 2$) *and* $g\in \mathfrak{PI}^{p}( \mathbb{R})\cap \mathfrak{PI}^{p^{\prime }}(\mathbb{R})$. *If*

3.5 $$ \bigl(I^{-}\operatorname{supp}\hat{h}\bigr)\cap \operatorname{supp}\hat{g}=\emptyset, $$

*where* $I^{-}=[-1,0)$, *then the Poisson inequality* $\mathfrak{PI}(hg) \leq h\mathfrak{PI}g$ *holds*.

### Proof

By condition (3.5), we obtain that (see [4])

$$ (t\operatorname{supp}\hat{h})\cap \operatorname{supp}\hat{g}=\emptyset $$

for any $t \in I^{-}$, which is equivalent to

$$ \operatorname{supp}\hat{h}\biggl(\frac{.}{t}\biggr)\cap \operatorname{supp}\hat{g}= \emptyset $$

for any $t \in I^{-}$.

By the embedding theorem and Hölder’s inequality, we obtain

3.6 $$ \begin{aligned}[b] & \int_{A_{k_{j+1},j+1}}\bigl(h(u)-k_{j+1}\bigr)_{+}\,dx \\ &\quad \leq \biggl( \int_{A_{k_{j+1},j}}\bigl(\bigl(h(u)-k_{j+1}\bigr)_{+} \zeta_{j}^{q}\bigr)^{\frac{n}{n-1}}\,dx \biggr)^{\frac{n-1}{n}} \vert A_{k_{j+1},j+1}\vert ^{1/n} \\ &\quad \leq \gamma \int_{A_{k_{j+1},j}}\bigl\vert \nabla \bigl(\bigl(h(u)-k_{j+1} \bigr)_{+}\zeta_{j}^{q}\bigr)\bigr\vert \vert A_{k_{j+1},j}\vert ^{1/n} \\ &\quad \leq \gamma \biggl( \int_{A_{k_{j+1},j}}g(u)\vert \nabla u\vert \zeta_{j}^{q} \,dx \\ &\qquad {} + \int_{A_{k_{j+1},j}}\bigl(h(u)-k_{j+1}\bigr)_{+} \vert \nabla \zeta_{j}\vert \zeta_{j}^{q-1}\,dx \biggr) \vert A_{k_{j+1},j}\vert ^{1/n}. \end{aligned} $$

Let $\ell =\delta (\rho)/\rho $. We estimate the first term on the right-hand side of (3.6) as follows:

3.7 $$ \begin{aligned}[b] & \int_{A_{k_{j+1},j}}g(u)\vert \nabla u\vert \zeta_{j}^{q} \,dx \\ &\quad =\frac{1}{g( \ell)} \int_{A_{k_{j+1},j}}g(u)g(\ell)\vert \nabla u\vert \zeta_{j}^{q}\,dx \\ & \quad \leq \ell \int_{A_{k_{j+1},j}}g(u)\zeta_{j}^{q}\,dx + \frac{1}{g(\ell)} \int_{A_{k_{j+1},j}}g(u)G\bigl(\vert \nabla u\vert \bigr) \zeta_{j}^{q}\,dx \\ &\quad \leq 2^{j}\frac{ \ell }{k} \int_{A_{k_{j+1},j}}\bigl(h(u)-k_{j+1}\bigr)_{+}g(u) \zeta_{j}^{q}\,dx +\frac{1}{g( \ell)} \int_{A_{k_{j+1},j}}g(u)G\bigl(\vert \nabla u\vert \bigr) \zeta_{j}^{q}\,dx. \end{aligned} $$

It follows that

3.8 $$ \begin{aligned}[b] & \int_{A_{k_{j+1},j}}g(u)\vert \nabla u\vert \zeta_{j}^{q} \,dx \\ &\quad \leq \gamma (1- \varrho)^{-\gamma }2^{j\gamma } \biggl( \frac{\ell }{k}+\frac{1}{g( \ell)} \biggr)\rho^{-1} g \biggl( \frac{\delta (\rho)}{\rho } \biggr) \int_{A_{k_{j},j}}\bigl(h(u)-k_{j}\bigr)_{+}\,dx \end{aligned} $$

from the previous inequality and Lemma 2.2.

Since

3.9 $$ k\geq G(\ell)=G \biggl( \frac{\delta (\rho)}{\rho } \biggr), $$

we obtain

3.10 $$ y_{j+1}= \int_{A_{k_{j+1},j+1}}\bigl(h(u)-k_{j+1}\bigr)\,dx \leq \gamma (1- \varrho)^{-\gamma }2^{j\gamma }\rho^{-1} k^{-\frac{1}{n}}y_{j}^{1+ \frac{1}{n}} $$

from (3.6) and (3.7), which gives that

3.11 $$ k\geq \gamma (1-\varrho)^{-\gamma }\rho^{-n} \int_{B_{\frac{1-\varrho }{2}\rho }(\bar{x})}h(u)\,dx. $$

(3.8) and (3.9) also imply that

3.12 $$ h\bigl(u(\bar{x})\bigr)\leq \gamma (1-\varrho)^{-\gamma }G \biggl( \frac{\delta ( \rho)}{\rho } \biggr) + \gamma (1-\varrho)^{-\gamma } \rho^{-n} \int_{B_{\frac{1-\varrho }{2}\rho }(\bar{x})} h(u)\,dx. $$

Since

$$\begin{aligned} \int_{B_{\frac{1-\varrho }{2}\rho }(\bar{x})}h(u)\,dx &\leq \delta ( \rho) \int_{B_{(1-\varrho)\rho }(\bar{x})}g(u)\xi^{q}\,dx \\ &\leq \gamma (1-\varrho)^{-1}\frac{\delta (\rho)}{\rho } \int_{B_{(1-\varrho)\rho }(\bar{x})}g\bigl(\vert \nabla u\vert \bigr) \xi^{q-1}\,dx, \end{aligned}$$

we obtain that

3.13 $$ \begin{aligned}[b] \int_{B_{\frac{1-\varrho }{2}\rho }(\bar{x})}h(u)\,dx\leq{}& \gamma (1- \varrho)^{-1} \frac{\delta (\rho)}{M(\rho)} \int_{B_{(1-\varrho)\rho }(\bar{x})}G\bigl(\vert \nabla u\vert \bigr) \xi^{q}\,dx \\ &{} + \gamma (1-\varrho)^{-1}\frac{\delta (\rho)}{\rho } g \biggl( \frac{M( \rho)}{\rho } \biggr) \rho^{n} \end{aligned} $$

and

3.14 $$ \int_{B_{(1-\varrho)\rho }(\bar{x})}G\bigl(\vert \nabla u\vert \bigr) \xi^{q}\,dx \leq \gamma (1-\varrho)^{-\gamma }G \biggl( \frac{M(\rho)}{\rho } \biggr) \rho ^{n}. $$

Combining (3.13) and (3.14), we have

3.15 $$ \int_{B_{(1-\varrho)\rho }(\bar{x})}h(u)\,dx \leq \gamma (1-\varrho)^{- \gamma } \frac{\delta (\rho)}{\rho } g \biggl( \frac{M(\rho)}{\rho } \biggr) \rho^{n}. $$

As a consequence, (3.1) holds. Thus, by invoking Theorem 3.1, the Radon transform of function *hg* satisfies the Poisson inequality

$$ \mathfrak{PI}(hg)\leq h\mathfrak{PI}g. $$

 □

## Conclusions

This paper was mainly devoted to studying a new Poisson inequality for the Radon transform of infinitely differentiable functions. An application of it was also given.

## References

[1] Bedrosian E. (1963). A product theorem for Hilbert transform. Proc. IEEE.

[2] Brown J. (1974). Analytic signals and product theorems for Hilbert transforms. IEEE Trans. Circuits Syst..

[3] Brown J. (1986). A Hilbert transform product theorem. Proc. IEEE.

[4] Carleman T. (1923). Über die approximation analytischer funktionen durch lineare aggregate von vorgegebenen potenzen. Ark. Mat. Astron. Fys..

[5] Cohen L. (1995). Time-Frequency Analysis: Theory and Applications.

[6] Donoghue W. (1969). Distributions and Fourier Transforms.

[7] Gabor D. (1946). Theory of communication. J. Inst. Electr. Eng..

[8] Gasquet C., Witomski P. (1999). Fourier Analysis and Applications.

[9] Huang J. (1980). A new verification rule and its applications. IEEE Trans. Softw. Eng..

[10] Kokilashvili, V.: Singular operators in weighted spaces. In: Functions, Seres, Operators. Colloquia Mathematica Societatis Janos Bolyai, vol. 35. Budapest (1980)

[11] Krámli A., Pergel J. (1975). On the Radon–Nikodým derivatives of measures generated by means of processes of autoregression type. Alkalmaz. Mat. Lapok.

[12] Krasichkov-Ternovskiĭ I.F. (1997). Estimates for the subharmonic difference of subharmonic functions. II. Mat. Sb..

[13] Levin B. (1980). Distribution of Zeros of Entire Functions.

[14] Levin B. (1996). Lectures on Entire Functions.

[15] Long P., Deng G. (2006). Generalized quasi-analyticity of infinitely differentiable functions with several complex variables on closed angular domain. J. Math. Res. Exposition.

[16] Luan K., Vieira J. (2017). Poisson-type inequalities for growth properties of positive superharmonic functions. J. Inequal. Appl..

[17] Nikol’skiĭ, N.K.: Selected problems of the weighted approximation and of spectral analysis. Trudy Mat. Inst. Steklov. **120** (1974) English transl. in Proc. Steklov Inst. Math., 120 (1976)

[18] Nuttall A., Bedrosian E. (1966). On the quadrature approximation for the Hilbert transform of modulated signals. Proc. IEEE.

[19] Oppenheim A., Lim J. (1981). The importance of phase in signal. Proc. IEEE.

[20] Pandey J. (1996). The Hilbert Transform of Schwartz Distributions and Applications.

[21] Ren Y., Yang P. (2013). Growth estimates for modified Neumann integrals in a half space. J. Inequal. Appl..

[22] Rosenstock H. (1968). Level touchings in a randon walk. SIAM J. Appl. Math..

[23] Xiao Y. (1989). A decomposition theorem for infinitely differentiable functions of two variables. Chin. Ann. Math., Ser. A.

[24] Yang D., Ren Y. (2014). Dirichlet problem on the upper half space. Proc. Indian Acad. Sci. Math. Sci..

